# On a Possible Unified Scaling Law for Volcanic Eruption Durations

**DOI:** 10.1038/srep22289

**Published:** 2016-03-01

**Authors:** Flavio Cannavò, Giuseppe Nunnari

**Affiliations:** 1 Istituto Nazionale di Geofisica e Vulcanologia, Osservatorio Etneo, Catania, 95123, Italy; 2 University of Catania, Dipartimento di Ingegneria Elettrica, Elettronica ed Informatica, Catania, 95125, Italy

## Abstract

Volcanoes constitute dissipative systems with many degrees of freedom. Their eruptions are the result of complex processes that involve interacting chemical-physical systems. At present, due to the complexity of involved phenomena and to the lack of precise measurements, both analytical and numerical models are unable to simultaneously include the main processes involved in eruptions thus making forecasts of volcanic dynamics rather unreliable. On the other hand, accurate forecasts of some eruption parameters, such as the duration, could be a key factor in natural hazard estimation and mitigation. Analyzing a large database with most of all the known volcanic eruptions, we have determined that the duration of eruptions seems to be described by a universal distribution which characterizes eruption duration dynamics. In particular, this paper presents a plausible global power-law distribution of durations of volcanic eruptions that holds worldwide for different volcanic environments. We also introduce a new, simple and realistic pipe model that can follow the same found empirical distribution. Since the proposed model belongs to the family of the self-organized systems it may support the hypothesis that simple mechanisms can lead naturally to the emergent complexity in volcanic behaviour.

How long a single eruption lasts is one of the most intriguing questions concerning volcano activity. We know that volcanic eruptions can last from less than a day to thousands of years. In 1977, the lava lake at Nyiragongo drained in less than an hour. By contrast, Stromboli has had a low-level of activity since 450 BC (about 2,400 years)^1^. Although the median duration of historic eruptions is ~8 weeks^1^, this value is largely meaningless because of its considerable variance and its non-normal distribution as we demonstrate in the following.

Important factors governing a single eruption are the volume of melt accumulated in the magma reservoir and its degree of over-pressure, but also the possible contribution of new magma from depth. An eruption will normally last until the local melt has been depleted, or until the pressure level of the gas inside the magma reservoir reaches the pre-eruption pressure conditions. Nonetheless, this is a rather simplified overview. The internal plumbing system of an active volcano can be quite intricate, though recent monitoring efforts involving geodetic, seismic and geochemical measurements are helping at improving the estimations of size, depth, and activity of magma chambers under active volcanoes^2^,^3^,^4^.

In recent decades, different researches have been conducted to study volcanism in term of stochastic models^5^,^6^,^7^,^8^, while others have tried to characterize the scaling properties and self-similarity of volcanic processes^9^,^10^,^11^,^12^ such as inter-event times^13^,^14^, eruption magnitudes^15^,^16^ and tephra produced by eruptions^17^. Some authors have even proposed models based on fluid-rock complex systems to support the hypothesis of emerging self-organization in volcano dynamics^9^,^18^. Some recent works have also attempted to forecast the duration of volcanic eruption from a probabilistic perspective^19^ or from analytical assumptions (as the publicized, but unpublished, example of a forecast of the duration of the recent Bárðarbunga Iceland eruption, see http://icelandreview.com/news/2014/10/13/volcanologist-predicts-eruptions-end-march (Date of access: 20/11/2015) where the forecast of duration was based on heuristic considerations about the rate of subsidence of the volcano caldera after the beginning of the eruption). Nevertheless, the forecast of eruption duration is still impaired by the many uncertainties we have in volcanic activity modeling.

The objective of this paper is to present some newly found invariance properties for volcanic activity in terms of eruption time scales. These properties refer to the invariance of the probability distributions under wide changes of temporal scales. In particular, we focus on volcanic eruptions exploring the possibility that their durations follow a power-law distribution, at least for moderate-to-long lasting eruptions.

Power-law relationships in natural systems^20^ are relevant because they have some intriguing features. They are scale-invariant^21^ and thus have the same statistical properties at any scale and are not associated with one characteristic scale. In practise, this means that there is no single correct scale for their analysis because the same principles are valid irrespective of the temporal, spatial or strength scale of analysis^22^, and also makes it technically wrong to apply traditional statistics based on variance and standard deviation (such as regression analysis) when dealing with power-law distributed phenomena. The power-law systems share the concept of “universality”: it has been shown that completely different systems show similar power-law behaviour on approaching a critical state^23^. Moreover, the indication of a power-law behaviour in some data cannot only explain specific kinds of dynamics that might govern the particular natural phenomenon, but, thanks to the universality, may also indicate a deep connection with other, apparently unrelated systems^24^. Several empirical and theoretical investigations have suggested that geophysical systems seem to operate near a critical state, which results in the pervasiveness of power-law behaviour in several descriptors of their dynamics^25^,^26^. Thus the analysis of power-law and scaling relationships for volcanic eruptions would help us identify general principles that apply across a wide range of scales and types of eruptions, revealing a possible existence of universal principles within the nature of volcanic systems.

In order to sustain our hypothesis of power law for volcanic eruption durations we analyzed all the durations of the known eruptions, avoiding any kind of classification. The data of eruption durations for all the known volcanoes in the world to the best of our knowledge were retrieved from the Smithsonian Institution’s Global Volcanism Program (GVP) database^1^. To date, the GVP catalogue represents the most comprehensive effort in documenting, understanding, and disseminating information about global volcanic activity. It aggregates data from volcanoes and their eruptions for the last 10000 years. For each of the 10415 eruptions, the catalogue reports the starting date to the best of knowledge and for ~40% of them also the ending date. Of the eruptions in the catalogue, 53% are known to the month and 41% to the day^1^. Some ancient eruptions are known only to the century and many only to the year. When possible, the catalogue reports the uncertainty associated with the start and/or end dates (~11% of data).

Discounting the eruptions with unknown duration and any ongoing ones, the database consists of 3841 documented eruptions. The maximum time resolution of the documented eruptions is to the day but about half of the data set shows a larger uncertainty. Because of the time resolution of the GVP catalogue, the eruption duration has been considered as a discrete-valued variable and all the analyses have been carried out considering this constraint.

## Results

For the purposes of this study, a power-law distribution is described by a probability density function (PDF) *p*(*τ*) such that


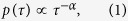


where the *p*(*τ*) represents the punctual probability that an eruption lasts *τ* > 0 days and *α* > 1 is the power-law exponent or scaling factor. For a power-law distributed variable, it is often useful to consider the complementary cumulative distribution function (CCDF) which will still be power-law distributed with:


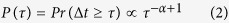


**Figure 1:**
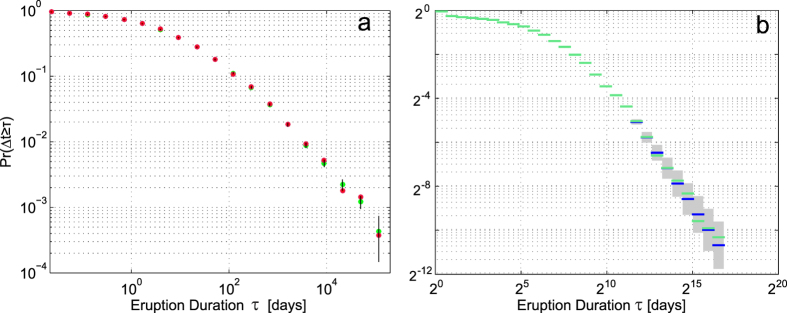
Empirical distribution of eruption durations. (**a**) Distribution for eruption durations expressed in days (red dots) and the associated uncertainty (black vertical line). The green dots represent the mean values. (**b**) Binned distribution of eruption durations (in green) with logarithmic binning scheme of 2^0.61*τ*^ and the fitted power-law (in blue) with the associated uncertainty (in gray).

In our analysis we considered the experimental CCDF of durations with its frequency-based approximation. As in most of the natural power-law-distributed systems, in the distribution of eruption durations (Fig. 1a), only the tail of the distribution follows a power-law and the Equations 1 and 2 apply only for values greater than some minimum *τ*_*min*_.

Figure 1a shows that, due to the found narrow error band, the distribution of durations seems to follow a power law, even when considering the associated duration uncertainties. In most of the studies of empirical distributions that follow power-laws the slope of the line (*α*, from Equation 1) is usually estimated by computing a least-squares linear regression on the log-log space^27^,^28^. Such graphical analyses can be grossly erroneous^29^,^30^ and thus their results may be unreliable. Following the trace in Clauset *et al.*^30^ we embraced the following statistically valid approach for investigating the hypothesis of power-law distribution for volcanic eruption durations: we first fitted the power law with a reliable method based on the maximum likelihood estimator (MLE), then tested the power-law’s plausibility and finally we compared the law against alternative distributions.

In order to take into account the uncertainties of the eruption durations, especially for the long and historic eruptions, we binned the data set by converting the values into a series of counts over a set of nonoverlapping ranges in duration size, and applied the methods in Virkar & Clauset^31^ for binned distributions to test our hypothesis.

**Figure 2:**
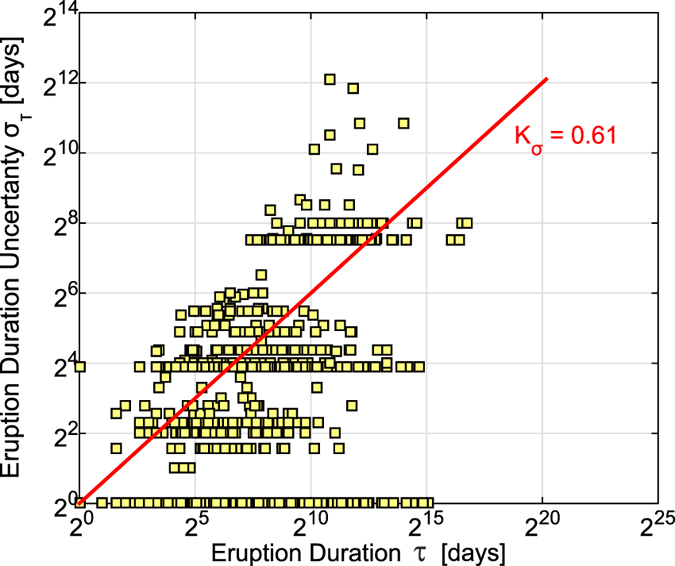
Uncertainty analysis. Scatter plot in log-log scale of eruption durations and associated uncertainties. The red line represents the best fit for the eruptions with uncertain duration and *K*_*σ*_ its angular coefficient.

By analyzing the scatter plot of the considered eruption durations against their uncertainties (see Fig. 2) we observed that for most of the eruptions the uncertainty increases with increasing duration and the log-log scale prompted us to choose the logarithmic binning scheme. Bearing in mind that increasing the coarseness of the binning scheme decreases the information available for estimation, and the required sample size increases^31^, we used a fine grained binning scheme (choosing a logarithm base of 2) in order to maximize the statistical accuracy of the analysis. With this choice, the critical sample size required to make the following tests statistically valid is minimum (a few hundreds of samples) as shown in Virkar & Clauset^31^. We roughly quantified the relationship between eruption durations and associated uncertainties with a linear regression in a log-log space, finding an angular coefficient of 0.61 that we used as a step to construct the logarithmic binning scheme. The binned CCDF was calculated by considering the counts of the durations over the bins (see Fig. 1b) thus losing part of the information but indirectly incorporating the uncertainty.

Before estimating the scaling parameter 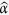, we needed to find the minimum duration *τ*_*min*_ that kept data for which the power-law model was valid. The adopted approach is based on the Kolmogorov-Smirnov (KS) distance^31^,^32^ to quantify the similarity of the estimated distribution and the theoretical power law. We found that for our data set the minimum KS distance is reached for *τ*_*min*_ = 1782 days. Assuming this value as *τ*_*min*_, we adopted the maximum-likelihood method for fitting the power-law model to the binned data and identifying the power-law exponent, obtaining 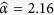. The uncertainties about *τ*_*min*_ and 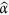 were computed by running 1000 Monte Carlo simulations obtaining a 1*σ*-uncertainty of 581 for *τ*_*min*_ and 0.22 for 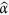. The number of eruptions with duration longer then *τ*_*min*_ is 189 distributed in 29 bins which are statistically sufficient quantities for empirical data to proceed with the power-law estimation^31^. Figure 1b shows the empirical binned CCDF for the eruption durations and the fitted binned power-law for *τ* ≥ *τ*_*min*_ and Table 1 shows the values of the found parameters.

Given the observed bin counts and a hypothesized power-law distribution from which the counts were drawn, we carried out a goodness-of-fit test in the form of a *p*-value to measure the likelihood that the hypothesized model would generate data with a more extreme deviation from the hypothesis than the empirical data^31^. The closer *p* is to 0 the more implausible is the model generating the data. Failing to reject the hypothesis is indeed a good indication of the hypothesis itself. As recommended in different works^30^,^31^ we used the conservative choice of ruling out the power-law if *p* < 0.1. The *p*-value, estimated by the KS statistic approach^31^, results to be 0.76. The rather high value of *p* does not necessarily imply the correctness of the power-law hypothesis, but confirms it as a valid hypothesis. In order to support the validity of our conjecture of power-law distribution, we also checked whether another similar kind of alternative heavy-tailed distribution might provide a better fit for our data. Although there are generally an unlimited number of alternative models which can fit the date with any desired precision, only a few are commonly proposed alternatives or correspond to common theoretical mechanisms^30^,^31^,^33^: the exponential^27^, the log-normal^34^ and the Weibull^35^ distributions. We compared the found power-law to the alternative distributions by using a likelihood ratio test for binned data^30^,^31^,^36^. The alternative distributions in their binned form were estimated by using an MLE approach^30^,^31^. Results in Table 1 show that the exponential distribution obtains a positive value of the index measuring the differences of the likelihoods in favour of the power-law distribution, 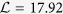, with an associated *p*-value of 0.00 which (being ≤0.1 thus rejecting the null hypothesis of similar likelihood for the compared distributions) allows us to be confident with the value of 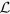 and (being 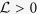) discard the alternative distribution. The comparisons with the log-normal and Weibull distributions give a *p*-value of 0.17 and 0.71 respectively, which (being >0.1) indicate that the sign of 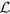 (although negative) is likely due to chance fluctuations around zero^36^ and thus not reliable, and the tests fail to favor one distribution over the other.

The assessment of *τ*_*min*_ relies on the quality of the catalogue and its completeness which is defined as the lowest duration at which 100% of the eruptions are likely detected. The high value of *τ*_*min*_ could be due to the lack of short eruptions in the historical catalogues and hence in a poor completeness of the GPV catalogue. Indeed, it is worth remarking that the GVP database is just a limited sample of recorded eruptions, and an undeterminable number of eruptions may have gone unnoticed or unrecorded entirely. The issue is even more complex since it is also very closely tied to defining the beginning and ending of eruptions. Any volcanic catalogue is clearly the result of observations recorded with complex, spatially and temporally heterogeneous techniques, and processed using a variety assumptions. Thus, even the best catalogues are heterogeneous and inconsistent in space and time because of the technical limitations in detecting eruptions, and are likely to show as many man-made changes in reporting as natural ones^1^. Choosing to analyze the GVP catalogue we have entirely espoused its assumptions, definitions and consequences. According to the GVP catalogue, an eruption following its predecessor by less than 3 months is considered as a phase of the same eruption, unless careful work has shown it to be distinct^1^,^37^.

**Figure 3:**
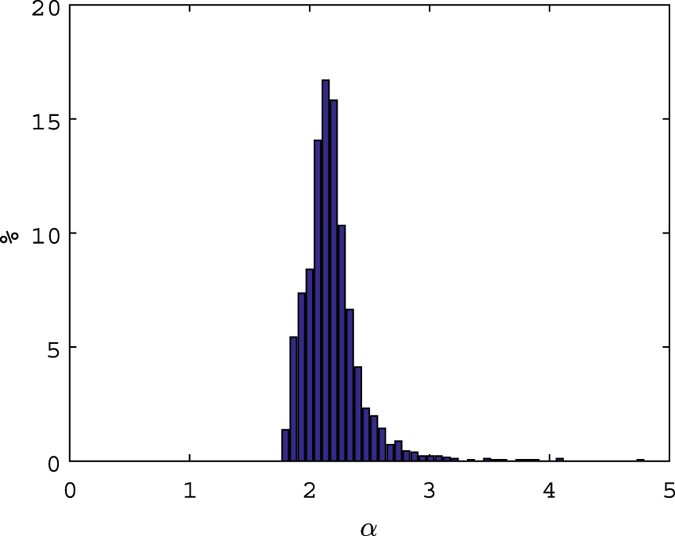
Normalized distribution of the estimated *α* parameter for the power laws obtained from Monte Carlo simulations.

In order to generalize our results we carried out a Monte Carlo simulation by randomly clusterizing a sub-set of eruptions and varying the binning scheme. To increase the result reliability, we selected only the clusters with a significant number of eruptions (i.e. ≥2000 that is the critical value which allows us to have a set of durations covering at least two decades with a probability of 95% for the used database). For each cluster of eruptions we carried out the analysis previously described by randomly varying the logarithmic binning scheme to check the power law distribution for the durations and to calculate its parameters. After 1000 simulations, the distribution of the found *α* parameter shows an expected value of 2.18 with a standard deviation of 0.25 (see Fig. 3) which matches the values estimated for the whole data set. We also analyzed the dependence of *α* with the upper duration of the catalogue through a scatterplot between the estimated *α* and the maximum duration for all the simulation (see Supplementary Fig. S3). The analysis does not show any first-order correlation between the maximum duration and the scaling factor, at least for large data sets.

**Figure 4:**
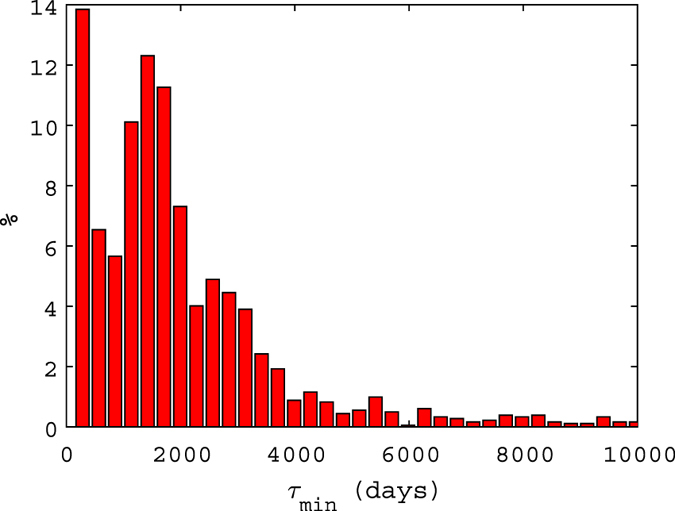
Normalized distribution of the estimated *τ*_*min*_ parameter for the power laws obtained from Monte Carlo simulations.

The distribution of the found *τ*_*min*_, shown in Fig. 4, though more scattered, has an expected value of 1895 days with a standard deviation of 1142, thus sparse but including the range obtained with the whole data set.

**Figure 5:**
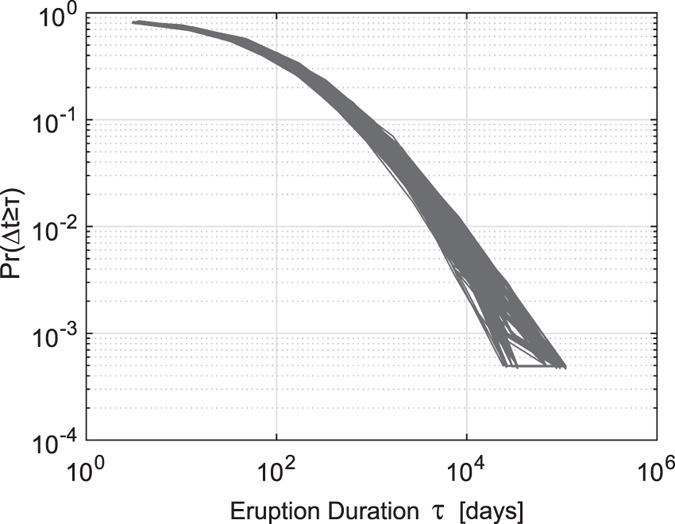
Distributions of eruption durations for 1000 Monte Carlo simulations. Each distribution is computed by considering all the eruptions of a randomly chosen set of volcanoes.

To check whether the found power-law in the distribution in Fig. 1 represents a limit of different distributions, or if it is a feature that holds for most volcanoes, we extended the analysis and considered clusterizing the eruptions for volcanoes. We conducted the same Monte Carlo simulations, but this time randomly selecting the volcanoes and taking all their eruptions in order to reach the critical number of eruptions of 2000. Results are comparable with those of the previous analysis thus confirming the previously found values (see Supplementary materials for details). For the same 1000 simulations, we plotted the empirical distributions of durations in Fig. 5, providing an idea of the dispersion of duration distributions around the found power-law and suggesting that the hypothesis of power-law distribution is independent from the volcano.

A further analysis performed on the single volcano with the highest number of confirmed eruptions (i.e. Mt. Etna, Italy with 151 eruptions) is presented in Supplementary materials. Although with different coefficients (*τ*_*min*_ = 154 and 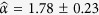), it confirms the power-law hypothesis. This allows stressing, that similarly to what happens for the Gutemberg-Ritcher relation, the power law may represent the universal mechanism which assumes different parameters for different areas.

It is worth noting that this analysis does not exclude that the duration distribution may not follow a power law for other clustering schemes of eruptions^38^. Moreover, a statistical limit of the found parameters is represented by the upper end of the duration scale, the tail of the distribution, that starts to come under the influence of the unavoidable duration truncation, at Holocene age (i.e. ~4 × 10^6^ days).

### A simple model of the volcanic process

**Figure 6:**
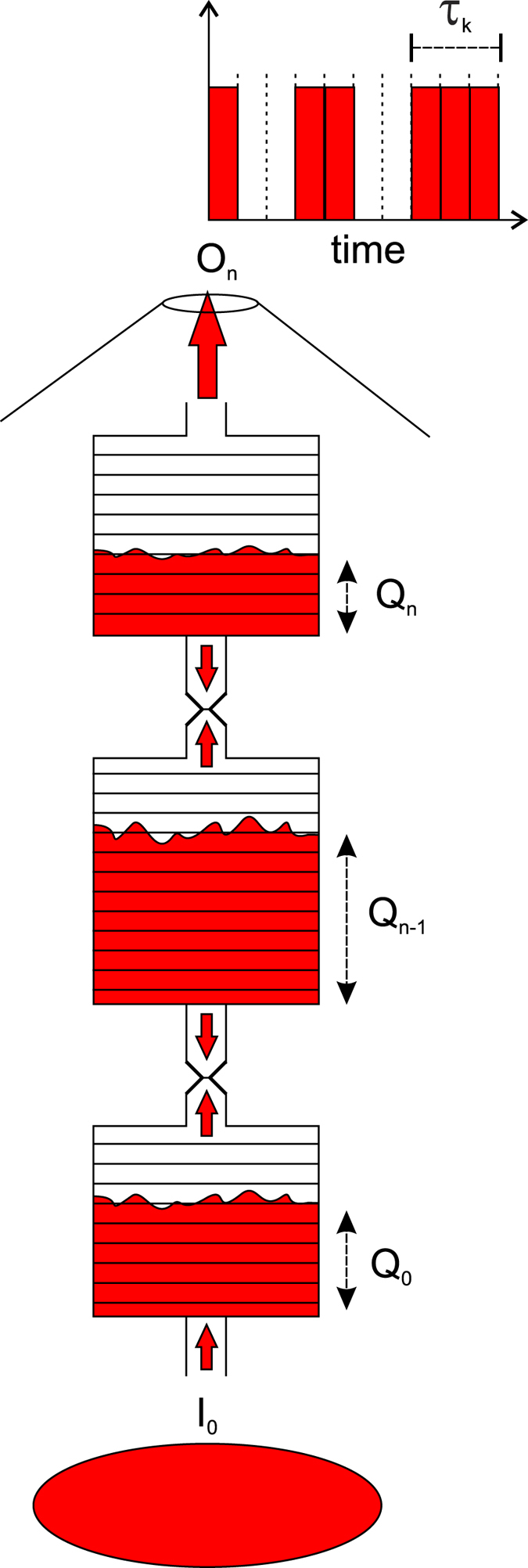
The proposed model to support the hypothesis of power-law distribution for time durations of volcanic eruptions. The fluxes between adjacent vessels are governed by stochastic processes. The uppermost vessel output represents eruptions. The eruption duration is calculated as the number of consecutive batch emissions from the uppermost vessel.

In the same way as the block-spring model for the earth crust and earthquakes^39^, we propose a simple model to try to replicate the same behaviour of eruption durations. This is qualitatively inspired on eruption mechanisms, represented by a pipe of *n* interconnected vessel cells, as sketched in Fig. 6. Each vessel stores an integer number of magma batches. Because of igneous differentiation^40^ and convective motions^41^, magma, despite generally having a net flux directed from bottom to top, depending on the physical conditions within a cell and in neighboring cells, may locally assume a downward motion. Thus, at each time step, part of the stored batches in a cell of the model can migrate to the upper cell or back to the lower cell. For this reason, each cell in the pipe (see Fig. 6), except the first and the last, is modeled as having two outputs: one directed upwards and the other directed downwards. The magma batches issuing from the top vessel, indicated as *O*_*n*_(*t*) and constrained up to one batch per time step, are considered as erupted. The purpose of the model is to compute the duration of eruptions, measured in time steps. Here, the duration is intended as the number of time steps during which the variable *O*_*n*_(*t*) is continuously positive. It is supposed that the lowest vessel is fed by a constant and continuous flux *I*_0_ of ascending magma batches. Thus the proposed model belongs to the class of slowly driven systems, which also include earthquakes^42^. In order to greatly simplify the model, it is supposed that the magma movement in the *i*_*th*_ cell is decided by local parameters as the local level of magma and a parameter *ρ*_*i*_. It is also supposed that *ρ*_*i*_ is a stochastic process uniformly distributed in the range [−1,1] and its strength represents the rate of magma batch transferring from the *i*_*th*_ cell to the neighboring cells. Furthermore, a threshold mechanism ensures that if the accumulated magma in a vessel exceeds the local minimal magma allocation (i.e. 1 batch) then at least one batch must necessarily rise up. The intrinsic nonlinearity in a threshold process should account for the slow-fast dynamics, which is one of the ingredients of self-organized criticality (SOC), as recognized in several natural systems, including earthquakes^42^. Formally, indicating as *Q*_*i*_(*t*) and *ϕ*_*i*_(*t*) the number of magma batches accumulated in the *i*_*th*_ vessel and the net flux of batches from the adjacent cells at the time *t*, respectively, the process in each model cell can be expressed as in Equation (3).


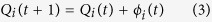


The net flux of batches can be computed as indicated in Equation (4)


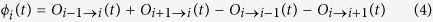


where *O*_*i*→*j*_ represents the magma batches that exit from the *i*–*th* vessel towards the *j*–*th* vessel, and are defined as:


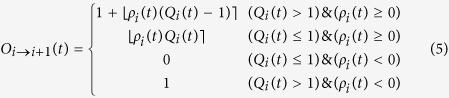


In expressions above, the symbols 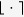 represent the rounding operator.


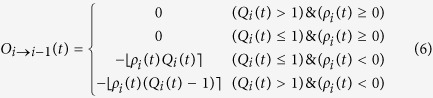


**Figure 7:**
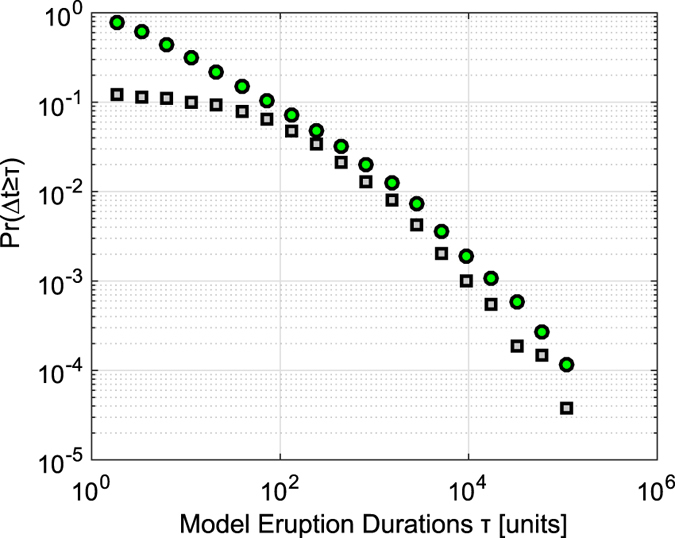
Power-law of the eruption durations for the simulated model (green circles) with *n* = 10000 and *N*_*T*_ = 10^7^ time steps. The scaling factor approaches to 2 by increasing *n* and *N*_*T*_. In gray squares the empirical power-law of eruption durations appropriately vertically translated for a visual comparison.

Expressions (5) and (6) clarify that the upward and downwards outputs are mutually exclusive, except in the case (*Q*_*i*_(*t*) > 1) & (*ρ*_*i*_(*t*) < 0). It can easily be verified, by noting that the mean values of *ρ*_*i*_(*t*) are null in Eqs (5) and (6), that the model output is directed from bottom to top on average. The general mathematical mechanism that rules the proposed model relies to the sandpile model^43^ constrained to an anisotropic 1-dimension with a privileged versus (towards the summit). For a sufficiently large number of vessels *n* (in our cases 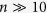) and for a sufficiently long time steps, the simulations of such a model show that the eruption durations follow a power-law distribution (see Fig. 7). Despite the extreme simplification, the scaling factor is close to the one obtained empirically from the real data (i.e. *α* → 2, see Fig. 7 for a visual comparison).

Thus, the proposed model seems to obey the same self-organizing (i.e. it always reaches the same attractor independently of the parameter values) behaviour of several other complex systems, that after the pioneering sandpile model^43^, has been recognized in several natural systems (e.g. earthquakes and forest fires^25^). The similarity of the models allows us to infer same mechanisms observed in other natural systems^44^. This class of systems shows a characteristic slow-fast dynamic which exhibits relatively small variations many times in the output and abrupt large changes a few times (but more than expected) in the output.

## Discussion

The results presented here suggest that the power-law behaviour in volcanic eruption durations seems a reasonably satisfactory representation of the duration-frequency distribution at least for moderate-to-long eruptions. The found power-law together with previous evidences for other volcanic observables^9^,^45^ could be the fingerprints that volcanoes self-organize to the critical state, thus supporting the hypothesis that the mechanisms behind an eruption belong to the class of SOC^26^. However, the induction of SOC nature for systems showing power-law behaviour is controversial, even among experts, and has attracted significant criticism^46^,^47^,^48^,^49^,^50^,^51^. Nevertheless, the origin of scaling law and SOC in natural systems is gaining increasingly interest in the field of statistical geophysics^47^,^52^,^53^,^54^,^55^,^56^. Recent studies have shown that the power-law behaviours attributable to SOC can be explained by a version of the central limit theorem applied to a sum of a large number of independent random variables described by a family of statistical heavy-tailed distributions, which need not be power laws^50^,^56^,^57^,^58^. Thus, the stochastic or SOC origin of the power-law behaviour in natural systems is currently a matter of heated debate. In the hypothesis of SOC origin of the found power-law behaviour, the knowledge of the scaling factor could be used to make inferences about the underlying processes, to test mechanistic models, and to estimate and predict patterns and processes operating beyond the scope of the observed data. The proposed simple model could support the self-organized mechanism of the volcanic eruptions by which the emergence of complexity from simple local interactions can be spontaneous, and therefore plausible as a source of natural complexity. The model also shows that a simple magma transfer/injection in some part of the magmatic system can cause a large variety of eruption durations. The hypothesis of SOC behaviour would lead to some intriguing consequences. First, a significant part of active volcanoes should be close to eruption instability, thus susceptible to erupt, though quiescent at the moment. The quantitative determination of the susceptible magma transfer/injection cannot be ascertained with precision, however the SOC behaviour would allow us to observe that the sensitive part of a volcano can be activated with relatively small perturbations. Second, we should await long eruptions more frequently than expected. This information should be relevant for volcanic hazard mitigation. Third, volcanic systems should show the emergence of invariant quantities. We have found that the volcanic eruption durations seem to show an exponent of the power-law that assumes globally the same value of the b-coefficient of the well-known Gutenber-Richter power-law for earthquakes^59^,^60^. Volcanic systems and earthquakes seem thus to share the same fundamental SOC behaviour whereby the systems naturally and independently of the physical parameters reach a critical state which can abruptly change for stochastic low-powered reasons (thus unpredictable with feasible precision).

In light of the found characteristic of volcanic eruption durations, and due to the enormous quantities of unknowns and the considerable uncertainties in the estimated parameters of the Earth and volcanoes interior, which are modeled as stochastic processes in the proposed model, we believe that the forecasts of eruption durations in terms of statistical expectation have the same reliability as for the earthquake magnitude forecast.

However, we would underline that the proposed analysis is intended as exploratory rather than explanatory. It is clearly tied to the limits of the catalogue, to the long time coverage, thus to indirect and partially inaccurate measurements, as well as the intrinsic heterogeneities in eruption definition.

Notwithstanding, similarly to the seismological literature, the found relation could be a reasonably satisfactory representation of the volcanic duration-frequency distribution of large populations of eruptions in terms of moderate-to-long lasting events, which could break down in the tails of very short or very long cases. Further studies and evidences are needed to confirm or refute the proposed hypothesis.

## Methods

The uncertainties associated to the durations were calculated as the sums of the uncertainties (given explicitly or with date truncation) of the relative start and end dates. For the calculation of the error band in the empirical distribution of eruption durations (in Fig. 1a) we used a bootstrap resampling for propagating the duration uncertainties. In this work, we adopted the reliable method based on the maximum likelihood estimator (MLE) to estimate the power-law exponent. MLE determines the unknown exponent that maximizes the likelihood of the power-law given the observed data.

In order to choose the value of *τ*_*min*_ that makes the binned CCDF distributions of the best-fit power-law model and the measured data as similar as possible above *τ*_*min*_, we used the method described in Virkar & Clauset^31^. To quantify the similarity of the two probability distributions, we used the Kolmogorov-Smirnov (KS) distance^32^, thus estimating the *τ*_*min*_ as the *τ* that minimizes such a distance for *τ* ≥ *τ*_*min*_. Then we used a nonparametric bootstrap method^61^ to estimate the error associated to *τ*_*min*_. For the Monte Carlo simulations used to estimate the power-law parameter distributions we varied the logarithmic binning scheme choosing the base randomly between 2 and 10.

We used a goodness-of-fit test generating a *p*-value that quantified the likelihood of the dataset, supposing our hypothesis to be true^30^. Such a test is based on measuring the distance between the empirical and the hypothesized distributions. This distance is compared with a set of distances calculated for comparable synthetic data sets obtained from the same distribution, and the *p*-value corresponds to the fraction of the synthetic distances that are larger than the empirical distance. In order to obtain accurate estimates of *p*-value, we looked for synthetic data that showed a distribution similar to the empirical data below *τ*_*min*_ but that followed the fitted power-law with scaling parameter 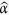 above *τ*_*min*_. We fitted each synthetic data set separately to its own power-law model and calculated the KS distance for each one relative to its own model. To generate such datasets, we adopted a semiparametric approach described in Clauset *et al.*^30^. We generated a total of 1000 synthetic data sets.

We evaluated the power-law distribution against another distribution by using the log-likelihood ratio test^33^,^62^ which compares the likelihood of the data under two alternative distributions with PDFs *p*_1_(*τ*) and *p*_2_(*τ*) by calculating the index in (7).


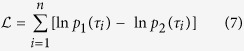


A positive value of 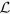 suggests a better fit for distribution *p*_1_ against *p*_2_. The test cannot indicate anything if the expected value of 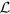 fluctuates close to zero. Thus we look at the probability that the measured log-likelihood ratio is equal or larger than the observed value 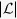 given by^36^


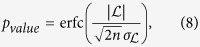


where erfc is the complementary Gaussian error function. This *p*-value represents the probability that we measure a given value of 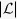 when the true value of 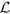 is unreliable for discriminating between the distribution (its expected value is close to zero). If this *p*-value is small (usually, *p* ≤ 0.1), then the calculated 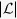 is unlikely to be a random result and thus its information is statistically valid and it is possible to discriminate between the two distributions^63^.

In the proposed discrete model simulations we used *I*_0_ = 1, but the model shows the same behaviour for any other parameter values and thus it is justified a vertical translation, in order to compare the two distributions shown in Fig. 7.

## Additional Information

**How to cite this article**: Cannavò, F. and Nunnari, G. On a Possible Unified Scaling Law for Volcanic Eruption Durations. *Sci. Rep.* **6**, 22289; doi: 10.1038/srep22289 (2016).

## Supplementary Material

Supplementary Information

## Figures and Tables

**Table 1:** Parameters of the fitted power-law, their *p* values and their comparison against the three considered alternatives.

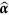	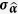	*τ*_*min*_ [days]	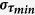 [days]	Power-law *p*	Log-normal		Exponential		Weibull		#{*τ* > *τ*_*min*_}
					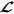	*p*	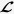	*p*	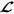	*p*	
2.16	0.22	1782	581	0.76	−1.36	0.17	17.92	0.00	−0.37	0.71	189

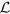
 is the normalized log-likelihood ratio against alternatives (positive indicates that the power-law is favored over the alternative) and the *p*-value for the significance of the test (the test is considered significant only if *p* < 0.1 and indicative only with 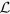 diverging from 0).
