# Supplementary material for: On a Possible Unified Scaling Law for Volcanic Eruption Durations
Source: Sci Rep. 2016 Mar 1;6:22289. doi: 10.1038/srep22289 (PMC4772095; doi:10.1038/srep22289)
Supplement: Supplementary Information [file srep22289-s1.pdf]

On a Possible Unified Scaling Law for Volcanic Eruption Durations

Flavio Cannavò[1,*] and Giuseppe Nunnari[2]

[1] *Istituto Nazionale di Geofisica e Vulcanologia, Osservatorio Etneo, Catania, 95123, Italy*
[2] *University of Catania, Dipartimento di Ingegneria Elettrica, Elettronica ed Informatica, Catania, 95125, Italy*

[*] flavio.cannavo@ingv.it

Supplementary

We analyzed a single volcano eruptions to support our hypothesis of power-law distribution from local to global scale. We chose Mt. Etna (Italy) volcano because its highest number of confirmed eruption (196) in the GVP catalogue. Figure S1 shows the distribution of the eruption durations for Mt. Etna in a log-log plot.

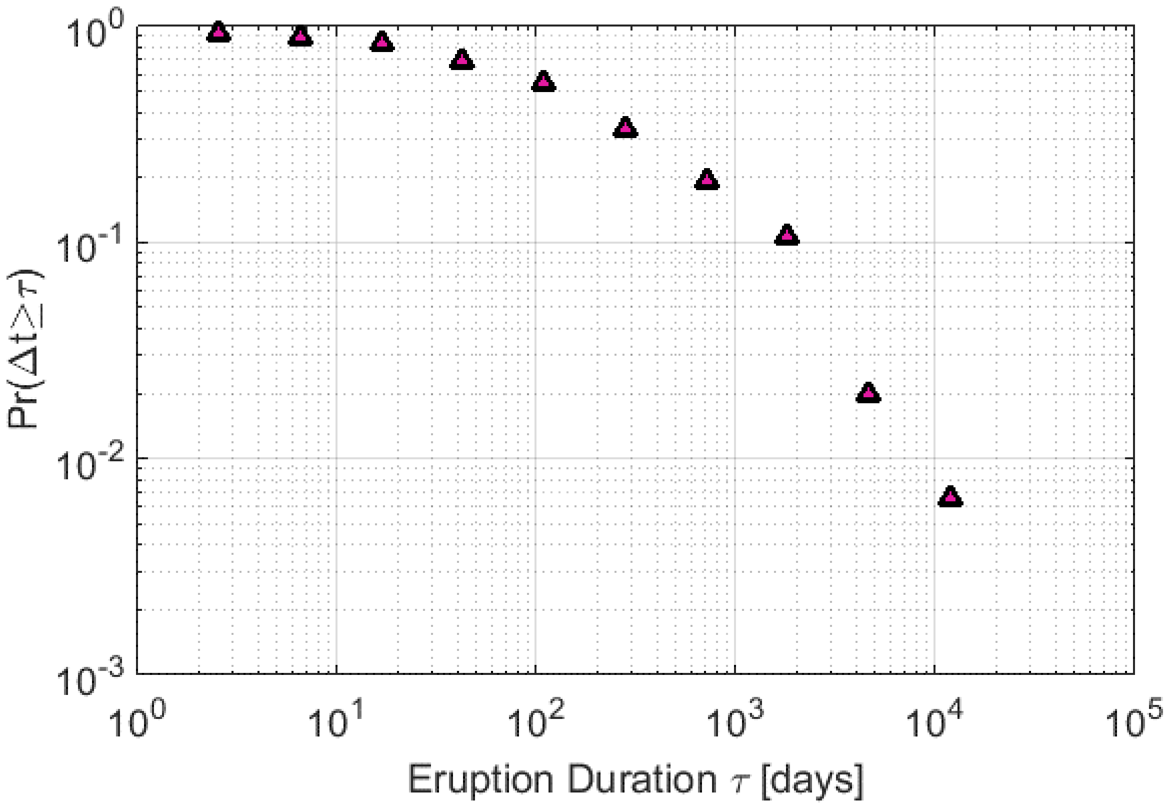

Supplementary Figure S1. CDF of the eruption durations for Mt. Etna

We carried out the same analyses of the empirical distribution reported in the paper. We found a $\tau_{min} = 154$ and $\hat{\alpha} = 1.78 \pm 0.23$. For the goodness-of-fit test the p-values is 0.14 thus above our threshold of 0.1, meaning that the power-law hypothesis is not ruled out by our test. Table 1 shows the comparison of the fitted power-law against alternative distributions. It is worth noting that although the comparison with the stretched exponential is slightly in favor of this, the very small size of the number of eruptions in the tail of the distribution ($\#\{\tau \geq \tau_{min}\}$=65) and the proximity of the p-value to 0.1 do not allow us, again, to reject the power-law hypothesis.

Supplementary Table S1. *Power-law's p value and comparison of the fitted power-law behavior against alternatives. $\mathscr{L}$ is the normalized log-likelihood ratio against alternatives (positive indicates that the power-law is favored over the alternative) and the p-value for the significance of the test (the test is considered significant only if p<0.1)*

Power-law *p*	Log-normal		Exponential		Stretched exponential	
	$\mathscr{L}$	*p*	$\mathscr{L}$	*p*	$\mathscr{L}$	*p*
0.139	-1.58	0.11	1.47	0.14	-1.69	0.09

To check if the found power-law for the whole data set represents a limit of different distributions, or it is a feature that holds for most of volcanos we extended the analysis and considered to clusterize the eruptions for volcanoes. We conducted Monte Carlo simulations by selecting randomly the volcanoes in order to reach the critical number of eruptions of 2000. After 10'000 simulations, the distribution of the found α parameter shows an expected value of 2.05 with a standard deviation of 0.19 (see Fig. S2) which is in agreement with the values estimated for the whole data set. The distribution of the found τ_{min}, showed in Fig. S2, though with two prominent peaks, has an expected value of 1125 days with a standard deviation of 872 thus overlapping the ranges obtained with the whole data set.

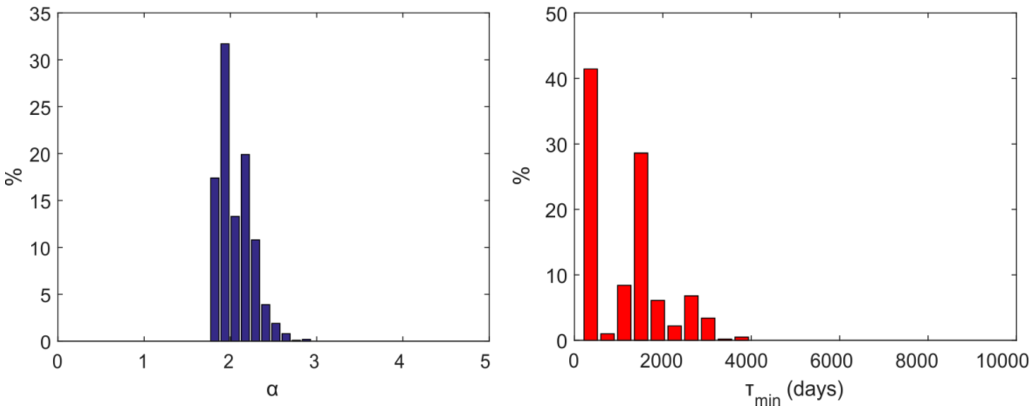

Supplementary Figure S2 Distributions of *α* (left panel) and τ_{min} (right panel) for the Monte Carlo simulations with clusterized volcanoes

We also analyzed the dependence of α with the upper duration of the catalogue through a scatterplot between the estimated α and the maximum duration for all the simulations (see Fig. S3). The analysis does not show any first-order correlation between the maximum duration and the scaling factor. In fact a regression analysis gives a value of $R^2 = 0.07$.

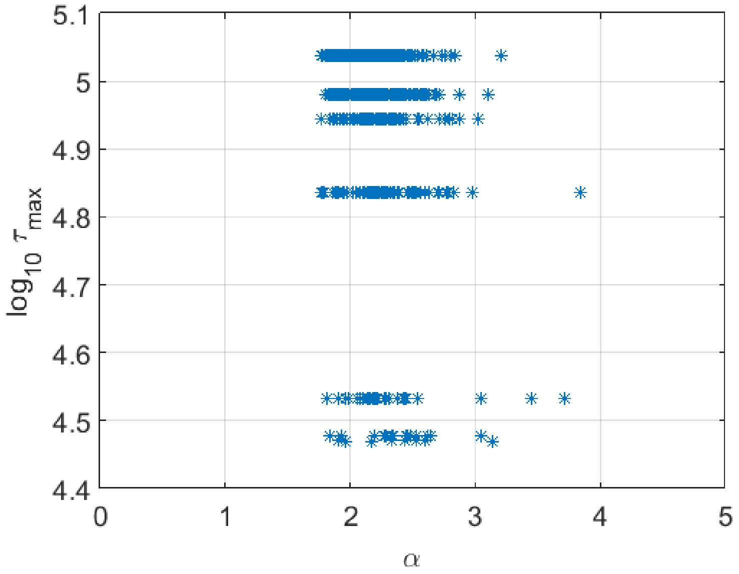

Supplementary Figure S3 Scatterplot between the estimated α and the maximum duration for all the simulations
